# Fluid Therapy During Cardiopulmonary Resuscitation

**DOI:** 10.3389/fvets.2020.625361

**Published:** 2021-01-28

**Authors:** Daniel J. Fletcher, Manuel Boller

**Affiliations:** ^1^Department of Clinical Sciences, College of Veterinary Medicine, Cornell University, Ithaca, NY, United States; ^2^Faculty of Veterinary and Agricultural Sciences, Melbourne Veterinary School, University of Melbourne, Werribee, VIC, Australia

**Keywords:** cardiopulmonary resuscitation, fluid resusciation, bicarbonate, lipid emulsion infusion, dogs, cats, calcium gluconate

## Abstract

Cardiopulmonary arrest (CPA), the acute cessation of blood flow and ventilation, is fatal if left untreated. Cardiopulmonary resuscitation (CPR) is targeted at restoring oxygen delivery to tissues to mitigate ischemic injury and to provide energy substrate to the tissues in order to achieve return of spontaneous circulation (ROSC). In addition to basic life support (BLS), targeted at replacing the mechanical aspects of circulation and ventilation, adjunctive advanced life support (ALS) interventions, such as intravenous fluid therapy, can improve the likelihood of ROSC depending on the specific characteristics of the patient. In hypovolemic patients with CPA, intravenous fluid boluses to improve preload and cardiac output are likely beneficial, and the use of hypertonic saline may confer additional neuroprotective effects. However, in euvolemic patients, isotonic or hypertonic crystalloid boluses may be detrimental due to decreased tissue blood flow caused by compromised tissue perfusion pressures. Synthetic colloids have not been shown to be beneficial in patients in CPA, and given their documented potential for harm, they are not recommended. Patients with documented electrolyte abnormalities such as hypokalemia or hyperkalemia benefit from therapy targeted at those disturbances, and patients with CPA induced by lipid soluble toxins may benefit from intravenous lipid emulsion therapy. Patients with prolonged CPA that have developed significant acidemia may benefit from intravenous buffer therapy, but patients with acute CPA may be harmed by buffers. In general, ALS fluid therapies should be used only if specific indications are present in the individual patient.

## Introduction

Untreated cardiopulmonary arrest (CPA), the acute cessation of blood flow and ventilation, is uniformly fatal, and the only known treatment to reverse it is cardiopulmonary resuscitation (CPR). CPR is delivered in two phases. The first and most important phase, basic life support (BLS), consists of chest compressions to re-establish blood flow and oxygen delivery to the tissues, and ventilation, to oxygenate the arterial blood and excrete carbon dioxide. The second phase of CPR, advanced life support (ALS), consists of adjunctive therapies that are targeted at improving the efficacy of the BLS interventions to maximize oxygen delivery to the tissues through drug therapy and defibrillation.

Intravenous fluid bolus therapy has been commonly used to increase preload during CPR in an attempt to increase cardiac output. In addition, specific fluid therapies to address acid-base and electrolyte disturbances that occur commonly in patients with CPA have been proposed. Finally, the use of intravenous lipid emulsions for the treatment of CPA secondary to specific toxicities has been studied. In 2012, the Reassessment Campaign on Veterinary Resuscitation (RECOVER) Initiative published the first evidence-based veterinary CPR guidelines after an extensive review of the primary literature (1). This manuscript summarizes the current evidence for and against the use of intravenous fluids, blood products, treatment of electrolyte disturbances, lipid emulsion and buffer therapy during CPR as well as the evidence-based RECOVER clinical guidelines related to these therapies.

## Resuscitative Fluid Therapy During CPR

### The Effect of Fluid Loading During CPR

**Figure 1:**
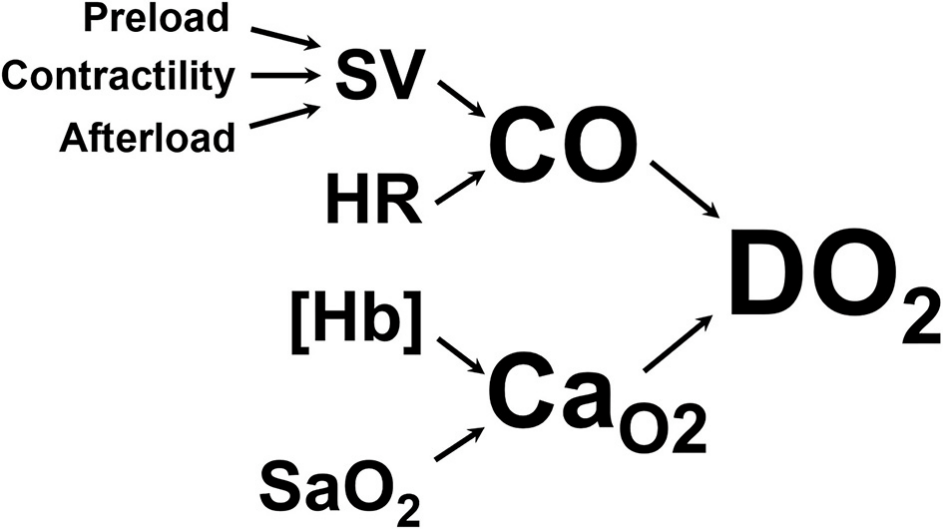
Oxygen delivery to the tissues (DO_2_) is the product of cardiac output (CO, the volume of blood ejected from the left ventricle into the aorta per minute) and arterial oxygen content (Ca_O2_, the volume of oxygen carried in the arterial blood). CO is the product of heart rate (HR) and stroke volume (SV, the volume of blood ejected from the left ventricle with each contraction). SV is determined by preload (the amount of blood in the left ventricle at the end of diastole), contractility (the force with which the left ventricle contracts), and afterload (the pressure against which the left ventricle has to push to get blood into the aorta). Ca_O2_ is determined by the amount of hemoglobin per unit of volume of blood ([Hb]) and the amount of that hemoglobin that is saturated with oxygen in the arterial blood (S_a_O_2_).

The primary goal of CPR is to restore oxygen delivery to tissues. Oxygen delivery is the product of cardiac output and arterial oxygen content, as shown in Figure 1. Basic life support targets both aspects of oxygen delivery by generating cardiac output through chest compressions and increasing arterial hemoglobin saturation through positive pressure ventilation. Once BLS is initiated, ALS interventions are targeted at enhancing oxygen delivery through various supplemental drug and defibrillation therapies, including intravenous fluid therapy.

**Figure 2:**
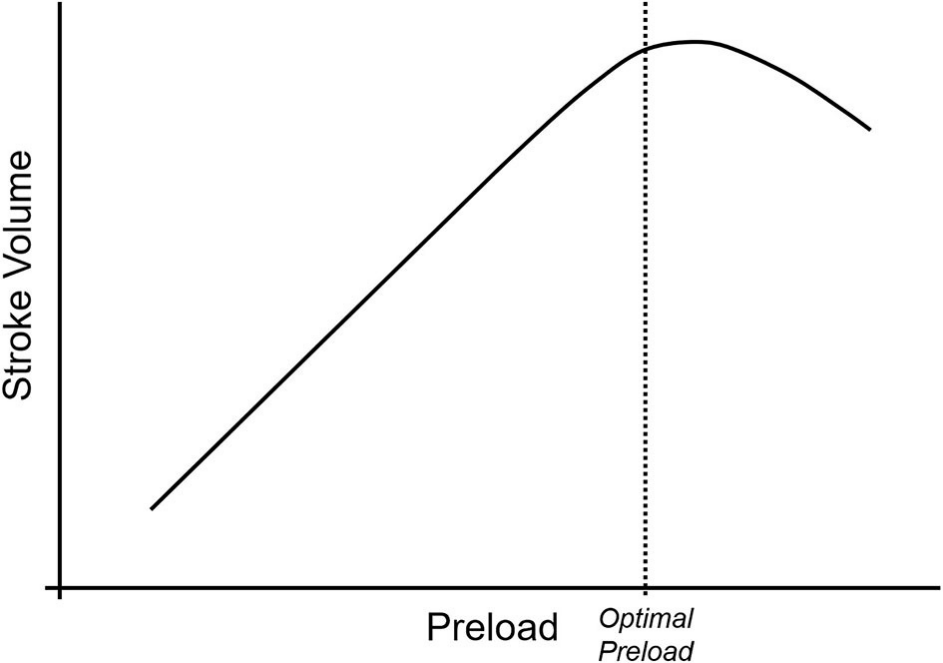
The Frank-Starling curve describes the relationship between preload (on the x-axis) and stroke volume (on the y-axis). At low preload, increases in the preload lead to linear increases in stroke volume due to increasing ventricular stretch. As the myocytes reach their maximum stretch capacity, the rate of stroke volume increase begins to drop with increasing preload, and as the myocytes become over-stretched, stroke volume begins to drop with increasing preload.

As demonstrated in Figure 1, cardiac output is the product of stroke volume and heart rate. One of the three determinants of stroke volume is preload, defined as end-diastolic ventricular wall tension or wall stretch, and clinically often represented as left ventricular end-diastolic volume or pressure (2). Preload can be increased in several ways, including through the infusion of intravenous fluids. However, the relationship between preload and stroke volume is not linear and is described by the Frank-Starling curve (Figure 2) (3). This shows that increasing a low preload leads to progressively increased stroke volume. This is because increased preload leads to left ventricular stretch, causing an increased contraction of the ventricle and improved stroke volume. After preload reaches and optimal volume, substantial further increases cause a diminished stroke volume, although this may largely be due to diastolic ventricular interaction rather than an overstretch of myocardial sarcomers (4).

Tissue perfusion is dependent on adequate cardiac output, but microvascular blood flow, and hence oxygen delivery to tissues is ultimately dependent on tissue perfusion pressures, which determine the amount of blood flow at the level of the tissue bed. The primary goal of CPR is to maintain perfusion of the core organs, the heart and the brain. In order to restore spontaneous circulation, restoration of blood flow and oxygen delivery to the myocardium is critical. Until adequate myocardial energy substrates are established, return of spontaneous circulation (ROSC) is unlikely. The coronary arteries arise from the base of the aorta just as it leaves the left ventricle and supply oxygen to the myocardium. Large epicardial vessels branch into smaller intramuscular vessels and ultimately to the myocardium before entering the capillary beds where oxygen delivery occurs. Venous drainage from the capillary beds feed into myocardial and then epicardial coronary veins. The latter ultimately coalesce into the coronary sinus, which drains into the right atrium. During systole, the myocardium contracts and compresses the myocardial coronary arteries, dramatically increasing resistance. Therefore, in the spontaneously beating heart, the bulk of myocardial perfusion occurs during ventricular diastole when the myocardium relaxes and myocardial coronary arterial resistance decreases. The driving pressure for blood flow into the myocardium during diastole is the aortic diastolic pressure (ADP). However, this driving pressure is resisted by the pressure within the left ventricle at the end of diastole (LVEDP) since the myocardial coronary arteries serving the left ventricle are exposed to left ventricular pressures as they course into the endocardium. During CPR, left ventricular end-diastolic pressures (i.e., the ventricular pressure during the relaxation phase of chest compressions) are very low, commonly less than the right atrial pressure (RAP), and coronary arterioles are maximally vasodilated (5). Therefore, the net force driving myocardial perfusion during CPR, termed coronary perfusion pressure (CoPP), is the difference between the driving pressure (aortic diastolic pressure) and the outflow pressure (right atrial diastolic pressure, RADP).

$$\begin{array}{l} \text{CoPP}=\text{ADP}-\text{RADP} \end{array}$$

**Figure 3:**
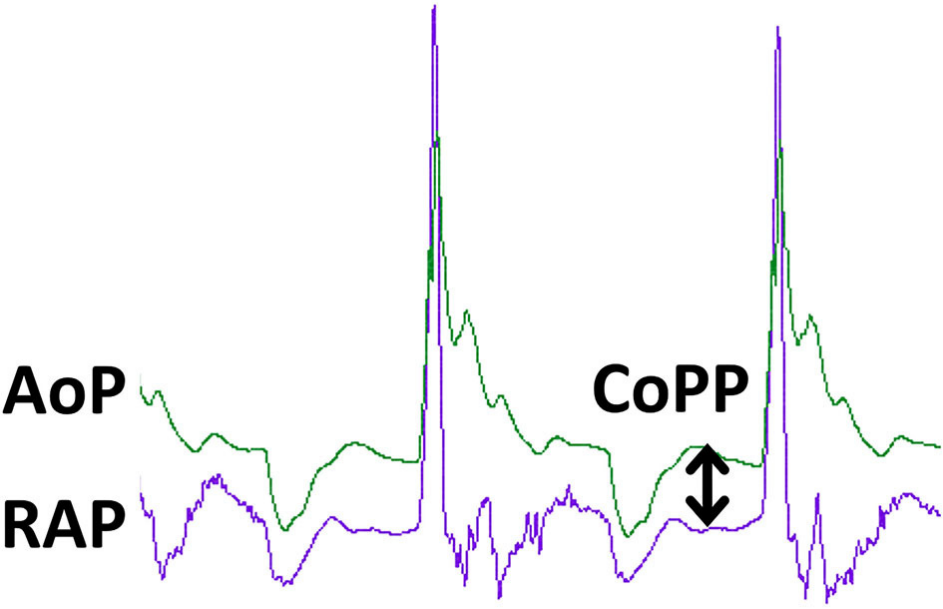
Pressure tracings during CPR in an experimental swine ventricular fibrillation model. The green tracing is aortic pressure (AoP) and the purple tracing is right atrial pressure (RAP). The peaks correspond to the compression phase of CPR. Coronary blood flow and oxygen delivery occur during the relaxation phase of chest compressions and is proportional to coronary perfusion pressure (CoPP), the difference between aortic pressure and right atrial pressure at the end of the relaxation phase, as denoted by the double arrowed vertical line. The tracing was obtained during mechanical, sternal chest compressions (100/min) delivered by the LUCAS (Lund University Cardiopulmonary Assist System) device to an anesthetized pig with VF arrest. Solid state pressure transducer catheters (Micro-Tip® Transducer, Millar Instruments, Houston, TX) were introduced through the left femoral artery and vein for continuous measurement of AoP and RAP.

Figure 3 shows aortic and right atrial pressure waveforms from a pig undergoing mechanical chest compressions. In this figure, the CoPP is calculated at the end of diastole, but other methods are used and this must be taken into consideration when comparing CoPP values across publications (6). Adequate CoPP has been shown to be critical for successful resuscitation from CPA in dogs. In early experimental studies using asphyxial CPA in dogs, it appeared that the CoPP required for ROSC was 30–40 mm Hg. This could not be achieved with chest compressions alone but required the addition of epinephrine (7). Further experimental studies in dogs conducted by Kern and colleagues in the 1980s demonstrated that a CoPP of at least 20 mm Hg led to ROSC, but 30 mm Hg was required for survival of more than 24 h (8). In an observational study in people including 100 adults with normothermic, non-traumatic CPA, non-survivors had a mean maximum CoPP of 8.4 mm Hg (SD 10 mm Hg), while those with ROSC achieved a mean maximum CoPP of 25.6 mm Hg (SD 7.7 mm Hg) (9).

Similarly, the pressure gradient leading to cerebral blood flow is cerebral perfusion pressure (CePP). The primary driving pressure for cerebral blood flow is mean arterial pressure (MAP), and the counteracting outflow pressure is either central venous pressure (CVP) or intracranial pressure (ICP), whichever is greater (CePP = MAP-CVP or MAP-ICP).

The dependence of tissue blood flow on perfusion pressures has a substantial impact on fluid therapy during CPR. Intravenous fluid loading has the potential to increase tissue driving pressures by increasing ADP and MAP during CPR. However, such fluid loading may also increase the counteracting outflow pressures, both RADP and CVP, which will decrease CoPP and CePP, respectively, and worsen oxygen delivery to myocardial and cerebral tissue beds. Ultimately, intravenous fluid loading should only be administered during CPR if it is likely that an increase in driving pressure (ADP or MAP) rather than in outflow pressure (RADP and CVP) will occur. By definition, this is more likely in patients that are effectively hypovolemic, either due to intravascular volume losses or inappropriate vasodilation (e.g., sepsis, SIRS, anaphylaxis). However, patients that are already fully fluid loaded or euvolemic will not likely experience significant increases in driving pressure. Rather, a further rise in intravascular volume in these patients will predominantly increase the outflow pressure (RADP and CVP), which will decrease perfusion pressures and result in decreased blood flow to the tissues.

These theoretical concerns have been documented in experimental studies. In a study of 18 euvolemic dogs with induced CPA, infusion of 11 ml/kg of either lactated Ringer's solution or whole blood resulted in a 34% increase in cardiac output, but a concurrent 26% decrease in left ventricular blood flow and a 35% decrease in cerebral blood flow. This was explained by a 22% reduction in CoPP that resulted from a disproportional increase in RADP compared to ADP (10). Similarly, Ditchey and Lindenfeld (11) found that volume loading during CPR in euvolemic dogs decreased cerebral and coronary blood flow due to increases in RADP and ICP that surpassed the increases in aortic systolic and diastolic pressures. In another study in euvolemic dogs with induced CPA, administration of intravenous fluid boluses in addition to epinephrine resulted in significant increases in aortic systolic and diastolic pressures and right atrial pressure, but no significant increase in myocardial blood flow compared to administration of epinephrine alone, suggesting that CoPP was not improved with fluid boluses in dogs receiving epinephrine (12). In euvolemic swine, the bolus administration of ~30 mL/kg of 0.9% saline reduced CoPP from 20.6 ± 8 mm Hg to 12.8 ± 5 mm Hg and significantly compromised ROSC (13). Unfortunately, to date there are no controlled, large-scale clinical trials evaluating the use of intravenous fluid boluses in euvolemic patients.

In patients with acute CPA, assessment of intravascular volume status can be challenging. Typical physical examination parameters that may be helpful, such as capillary refill time, mucous membrane color, heart rate and pulse quality, cannot be assessed in patients with CPA. Therefore, review of pre-arrest physical exam findings, an understanding of the precipitating causes of the arrest (e.g., a history of substantial gastrointestinal or urinary fluid losses or of anaphylaxis), or evidence of acute blood loss (e.g., documented external, cavity, or GI bleeding) are required to make the best intravenous fluid therapy plan during CPR. The RECOVER ALS guidelines recommend a conservative approach to fluid administration during CPR, reserving fluid boluses only for patients with known or suspected hypovolemia or distributive shock (1).

### Types of Resuscitation Fluids

As described previously, for hypovolemic patients in CPA, fluid boluses are recommended because they are likely to increase tissue driving pressures more than outflow pressures and improve CoPP and CePP. Generally, isotonic crystalloid fluid boluses of 1/4–1/3 of the total blood volume over 15–20 min (20–30 ml/kg in dogs, 10–20 ml/kg in cats) are recommended to treat hypovolemia.

There is experimental evidence that hypertonic saline (HS) may have beneficial effects beyond volume expansion during CPR. It is posited that HS may have neuroprotective effects by reducing cerebral edema, neuronal excitotoxicity, inflammation, and neuronal apoptosis. A recent experimental study in rats showed improved neurologic outcome post-CPR and reduced hippocampal apoptosis in rats receiving hypertonic saline during CPR compared to rats receiving isotonic crystalloids or synthetic colloids (14). Importantly, the study design did not include a control group that underwent CPR without fluid administration. An experimental study in cats in which CPR was started after 15 min of untreated VF, the investigators examined the effect of HS/hydroxyethyl starch (HES) (2 mL/kg IV) administered early during CPR compared to standard resuscitation without any fluid administration. The HS/HES combination resulted in a decreased CoPP and CePP during CPR compared to controls, but cats in the experimental group had significantly smaller volumes of frontal cortex no-reflow regions (areas of the brain in which there was no evidence of microvascular blood flow) 30 min after ROSC (15). A more recent study in swine compared administration of HS, HES, the combination of HS/HES (HHS), or normal saline (2 mL/kg IV once over 10 min) during open-chest CPR and showed that pigs in the HS and HHS group had increased myocardial blood flow and ROSC rates despite similar CoPP to the other 2 groups (16). Of note, a control group that received no fluids during CPR was lacking. Taken together, these studies suggest a potential neurologic benefit to administration of HS due to improved cerebral microcirculation and myocardial blood flow during CPR despite a possible decrease in CoPP and CePP, but the long-term outcome effects are unknown at this time.

There is limited clinical data available to inform a decision about the use of HS during CPR. A prospective, randomized controlled clinical trial (RCT) of 200 human patients with out-of-hospital cardiac arrest (OHCA) in which 100 patients received 2 ml/kg of HHS and 100 patients received 2 ml/kg of HES over 10 min showed no difference in short or long-term outcomes between the groups (17). A retrospective, registry-based study from Germany comparing human patients with OHCA who received either a HHS bolus at 2 ml/kg compared to a matched control group that received standard care without HHS showed improved survival to hospital admission with ROSC in the HHS group (18). However, long-term outcomes and survival to hospital discharge were not evaluated in this study. In addition, an accompanying editorial noted that short-term survival does not necessarily correlate with long-term survival or good functional outcome, that there are serious concerns about the safety of HES, and that there was no control for CPR quality in this retrospective study. These concerns suggest that more research is needed before this type of fluid resuscitation can be recommended routinely during CPR (19).

Taken together, this evidence suggests that HS may have a benefit over isotonic crystalloid fluids during CPR in patients with hypovolemia or vasodilatory shock. Given the consistent finding that intravenous fluid boluses of any kind lead to decreases in CoPP and CePP, and in the absence of any long-term outcome studies demonstrating a survival or neurologic function benefit to the use of HS during CPR in euvolemic patients, it is difficult to recommend volume expansion of any kind in patients known or suspected to be euvolemic during CPR. Taken in context with recent evidence of harm associated with the administration of HES (20) and the lack of any clinical or experimental evidence of benefit during or after CPR, the use of synthetic colloids alone or in combination with HS during CPR cannot be recommended.

Where CPA is caused or compounded by hypovolemia, fluid resuscitation is recommended as outlined above, and where hypovolemia is the consequence of severe hemorrhage, those fluids should include blood products (21). While there is very limited specific evidence to guide management of CPA due to exsanguination, it is reasonable to manage these cases similarly to patients with hemorrhagic shock not in CPA. A recent porcine study in which exsanguination cardiac arrest was induced by peripheral arterial hemorrhage, intra-arrest resuscitation with whole blood compared to 0.9% NaCl was significantly more effective in achieving ROSC (22). In people, a combination of fresh frozen plasma (FFP) and packed red blood cells (PRBC) at a ratio of 1:1 or 1:2 has been suggested, with the addition of cryoprecipitate and platelet concentrate as needed to prevent dilution of fibrinogen and platelets (23). In an RCT in people with hemorrhage after severe trauma, a lower relative amount of PRBC (FFP:PLT:PRBC = 1:1:1) compared to more PRBC (1:1:2) led to improved hemostasis, as more FFP and platelets were administered, and decreased death due to exsanguination but not overall mortality (24). Blood products can be administered by the intraosseus route (IO) initially until venous catheterization can be established. Studies in swine showed that administration of whole blood with a pressure bag inflated to 300 mm Hg will not lead to more hemolysis when blood is given by the IO route compared to a peripheral intravenous catheter, but that the infusion rate may be slower for IO (i.e., 40–100% of IV rate, depending on IO location) (25, 26).

Successful resuscitation from traumatic CPA is resource intensive. In addition to massive transfusion therapy, effective management of severe traumatic hemorrhage requires a comprehensive strategy potentially including damage control surgery, minimization of crystalloid administration, treatment of hyperfibrinolysis (e.g., tranexamic acid or aminocaproic acid), permissive hypotension and other interventions (27, 28). In people, many correctable causes for traumatic cardiac arrest require thoracotomy or thoracostomy (28). There are no studies published that specifically examine the use of blood transfusion during veterinary CPR or the causes, treatment and outcomes of traumatic CPA in dogs or cats. One veterinary case report describes the intra-arrest xenotransfusion of canine blood to a cat with severe anemia that ultimately survived to hospital discharge, an example for the potential benefit of intra-arrest red blood cell transfusions for reasons other than severe hemorrhage (29).

### Administration of Ice-Cold Fluids for Intra-Arrest or Early Post-Cardiac Arrest Cooling

Hypothermia protects all tissues, but in particular the brain, from the consequences of ischemia and reperfusion by a multitude of mechanisms (30). The post-cardiac arrest maintenance of body temperature below normal (i.e., targeted temperature management [TTM]) is currently recommended in humans that remain comatose with ROSC after out-of-hospital or in-hospital cardiac arrest (31). The 2012 RECOVER guidelines recommend that in dogs or cats that remain comatose after CPA, hypothermia to a core temperature of 32–34°C should be instituted as quickly as possible and maintained for 24–48 h (1). When considering all experimental animals studies involving rodents, swine, cats and dogs, hypothermia during the arrest has the most profound and long-lasting effect and any delay in hypothermia reduces the benefit (32–36). Thus, cooling that occurs during CPR and before ROSC might show benefit over post-cardiac arrest cooling alone. Animal experimental and human clinical studies evaluating intra-arrest therapeutic hypothermia explored selective brain cooling techniques (e.g., ice caps, nasopharyngeal cooling, or carotid flush) or systemic cooling approaches such as surface cooling with blankets, intravascular cooling catheters and intravenous administration of ice-cold fluids (34).

Experimental studies using intra-arrest cooling by ice-cold fluid bolus infusion are limited to swine (37, 38). In an OHCA model where ventricular fibrillation (VF) was left untreated for 8 min followed by CPR for at least 5 min before first defibrillation, more animals with intra-arrest cooling achieved ROSC (12/14) compared to those that were not cooled (6/14). The cooling group received a rapid intravenous bolus of 30 mL/kg of normal saline at 4°C during ongoing chest compressions, while the normothermic group received the same volume of saline at body temperature (37 ± 1°C). In these swine with a body weight of 27 ± 2.3 kg, the cold saline was effective in reducing the core body temperature to 34.2 ± 2.3°C within 5 min (37). In a second study, using a comparable VF model in similarly sized pigs, 30 mL/kg of acetated Ringer's solution at 4°C or at room temperature was infused over 22 min, starting early during CPR. With this slower administration regimen, the maximum temperature decrease achieved was only 1.62 ± 0.23°C in the cooling group compared to 1.14 ± 0.23°C in the control group, and the cold intra-arrest fluid conveyed no benefit on ROSC or survival to 3 h (38). These studies did not report any harm subsequent to fluid loading, but relevant assessments such as for presence or absence of pulmonary edema were not provided, and notably, control groups received the same amount of fluids. A further study in swine with VF subsequent to coronary occlusion demonstrated that the fluid loading with both ice-cold and room temperature saline during CPR (30 mL/kg) reduced CoPP by 40% when compared to no fluid administration (13). Thus, while the preponderance of animal studies supports intra-arrest cooling by a multitude of techniques and especially with prolonged CPA, evidence suggests that intra-arrest administration of large volumes of ice-cold saline is not beneficial in normovolemic animals (34). This could indicate that the benefits of cooling are offset by the harm of intra-arrest fluid loading.

A single randomized controlled trial in humans including 245 subjects with OHCA of any rhythm studied the effect of intra-arrest cooling with intravenous cold saline compared to no such cooling (39). Up to 2 L of ice-cold 0.9% saline were administered with a pressure bag, in combination with surface cooling with gel pads. It effectively reduced the core temperature and shortened the time to reach target temperature (34°C) by 75 min. However, intra-arrest cooling did not affect patient clinical outcome or surrogate markers of neurological injury. Likewise, a more recent non-RCT trial did not find any association between intra-arrest cooling of OHCA patients and important outcome measures such as sustained ROSC and survival to discharge (40).

Animal research suggests that early induction of hypothermia after ROSC is superior to delayed cooling (32). Intravenous administration of ice-cold isotonic saline represents an easy, rapid and cheap method to achieve this goal, and allows pre-hospital application in people. In swine, infusion of 4°C cold 0.9% saline at 30 mL/kg immediately after ROSC led to significantly improved short term neurological outcome compared to saline administration at room temperature (41). This cooling technique has otherwise not been specifically studied in animals but has been widely tested in people for almost 20 years. To date, six RCTs including a total of 2,500 subjects, did not show any benefit of early post-cardiac arrest cooling with rapid cold isotonic crystalloid infusion (42). Similar to the intra-arrest studies, boluses of up to 2 L of 0.9% saline, lactated Ringer's solution or Ringer's acetate at a rate of 100 mL/min were administered. Core temperature was ~1°C less than controls at hospital admission, but none of the studies showed improved favorable neurological outcome or survival to discharge. The largest study including 1,359 subjects demonstrated a significantly higher risk of re-arrest and occurrence of pulmonary edema in the pre-hospital cooling group (43). Thus, the routine administration of pre-hospital cooling with cold fluids is currently not recommended in people (31).

In summary, available evidence from animal studies suggest that mild hypothermia itself, either applied intra-arrest or early after ROSC mitigates ischemia-reperfusion injury. However, the harm associated with administration of large intravenous boluses of cold isotonic crystalloids during or after CPR suggests that other avenues for cooling should be considered in most cases. As dogs and cats are generally of lower thermal mass than humans, hypothermia (e.g., core temperature of 36°C or less) often occurs spontaneously during treatment of CPA and rapid administration of cold fluids is likely not necessary.

## Non-resuscitative Fluid Therapy During CPR

### Electrolyte Administration

Under special circumstances, the intra-arrest administration of calcium, magnesium, potassium and dextrose can be considered, but evidence of broad benefit is lacking.

Hyperkalemia can occur for a multitude of reasons and can lead to life-threatening cardiotoxicity and cardiopulmonary arrest once serum potassium concentrations are severely increased (e.g., >6.5 mEq/L) (44). Intravenous calcium administration should be considered when CPA is caused by hyperkalemia, in combination with other measures described for management of hyperkalemia-related cardiotoxicity and standard ALS interventions. Specifically, this includes infusion of 10% calcium gluconate (0.5–1 mL/kg, IV or IO) over 2–5 min, sodium bicarbonate (NaBic, 1 mEq/kg IV) over 2–5 min, and a mixture of regular insulin (0.5 U/kg) with 50% dextrose (2 g/kg) over 5 min intravenously (21). Calcium gluconate (0.5–1 mL/kg, IV or IO over 2–5 min) should further be given if CPA is attributed to hypocalcemia or to calcium channel blocker (CCB) overdose. For the latter, it has been recommended in people that calcium administration should be combined with lipid emulsion therapy (see below) (45). High dose insulin (e.g., bolus 0.5–2 U/kg followed by 0.5–2 U/kg/h infusion) together with dextrose in order to prevent hypoglycemia (e.g., 10–20% dextrose infusion) was also found to be effective in human case series of severe CCB poisoning and could be considered in people and animals (46–48). There is no indication in the literature that routine administration of calcium during CPR is beneficial, and hypercalcemia during reperfusion could be injurious by worsening post-ischemic cytoplasmatic calcium overload, although such evidence is experimental only (49–51). The 2012 RECOVER guidelines advise against routine administration of calcium during CPR but indicates that it may be considered in the presence of moderate to severe hypocalcemia (1).

As for calcium, there is no conclusive evidence for an overall benefit of routine administration of magnesium during cardiac arrest, despite several RCTs in people examining the utility of magnesium in OHCA and IHCA (52). Nevertheless, magnesium administration may be considered with some forms of pulseless ventricular tachycardia (pVT) (i.e., torsades de pointes), and possibly with pVT in general, or where it occurs in the presence of hypomagnesemia (46). In such situations, IV magnesium sulfate (15–25 mg/kg IV, bolus push) is recommended in humans, and similar doses have been suggested for dogs (12–40 mg/kg IV, slow bolus) (21, 53, 54).

Experimentally induced hypokalemia in dogs below 3.0 mEq/L was found to increase vulnerability to ventricular tachycardia and fibrillation through a variety of arrhythmia mechanisms (55–57). While expedient correction of the hypokalemia is reasonable, bolus administration of potassium during CPA is untested and not recommended (21). Instead, a potassium chloride CRI at 0.5 mEq/kg/hr should be considered.

### Intravenous Lipid Emulsions

The use of intravenous lipid emulsions (ILE) has been extensively reported in veterinary medicine to treat a wide range of poisonings and has recently been reviewed (58). In the context of CPA, it has been studied most extensively to reverse the effects of local anesthetic systemic toxicity (LAST), and a recent meta-analysis found ILE to improve survival in animal models including rats, rabbits, swine and dogs (59). In an experimental canine study, cardiac arrest was induced by rapid administration of bupivacaine (10 mg/kg) to isoflurane-anesthetized dogs. After 10 min of open-chest CPR, a 4 mL/kg bolus of a 20% lipid emulsion or 0.9% saline was followed by an infusion at 0.5 mL/kg/min for 10 min. All ILE treated dogs (6/6) and none of the saline treated dogs survived (60). There are many potential mechanisms of ILE's beneficial effects (61). The most popular theory is that ILE acts as a lipid sink and thus removes lipid soluble compounds from the target tissue, attenuating their toxic effect. In addition, there is some evidence that ILE elicits an additional cardiotonic response that is not explained by local anesthetic sequestration alone (62). Furthermore, studies in rats suggest that ILE may lessen ischemia-reperfusion injury by mitigating mitochondrial permeability transition (63, 64). Despite the lack of human RCTs or any clinical studies in veterinary species, the preponderance of the evidence suggests that ILE should be administered in dogs and cats with CPA caused by LAST.

The general recommendation for the clinical use of ILE in dogs and cats is (a) to administer 20% formulations, as 10% solutions are less effective; (b) to use inline filters (1.2 micron) as lipid aggregates are common; (c) to administer the solution as a bolus (1.5 mL/kg IV over 1 min) followed by an infusion (0.25 mL/kg/min IV CRI) for 30–60 min; (d) to limit the daily dose to 10 mL/kg (58). To facilitate administration during CPR, it appears reasonable to use repeat boluses of 1.5 mL/kg every second BLS cycle (i.e., every 4 min) instead of a constant rate infusion. Even though ILE might interfere with the hemodynamic response to epinephrine, it is currently not recommended in humans to adjust standard ALS measures (65).

Poisoning with other drugs than local anesthetics may also warrant the administration of ILE during CPR. Treatment with ILE is recommended in humans, and in extension animals, with CCB overdose alongside calcium gluconate, ALS measures as per standard guidelines and possibly other measures such as insulin/dextrose (45, 47). Two case reports including dogs with severe cardiotoxicity subsequent to CBB ingestion (i.e., diltiazem and lamotrigine) document successful use of ILE (48, 66). Unlike for CCB, the evidence for the benefit of ILE for the treatment of beta-blocker poisoning is conflicting (67). Human data consist of 1 observational study, 10 case series and 21 case reports, but inconsistent outcomes and co-administration of several other interventions disallow assessment for any benefit. The reports in people are complemented by 5 experimental studies in rabbits, none of which showed a significant treatment effect in support of ILE (67). The prospect of ILE as an effective antidote to other lipophilic compounds relevant to CPA, such as tricyclic antidepressants, may be considered on a case by case basis, following a similar ILE intra-arrest administration regimen as outlined for CCB above.

Adverse effects of ILE therapy, while infrequent, deserve mention (58). In a rabbit model of asphyxia CPA, ROSC rates with intralipid (3 mL/kg) compared to saline were significantly reduced (68). In one case report, a dog developed an acute respiratory distress-like syndrome after standard dose ILE administration (69). A systematic review further identified a long list of possible adverse effects in 27 animal and 87 human studies, including acute kidney injury, ventilation perfusion mismatch, acute lung injury, pancreatitis, hypersensitivity reactions, increased infection susceptibility, and CPA (70). In addition, the hyperlipidemia resulting from ILE therapy will compromise laboratory testing.

### Buffer Therapy

Clinical CPA causes profound acid-base abnormalities in dogs and cats, characterized by extreme venous acidemia with a pH as low as 6.6, venous hypercarbia that can reach beyond 100 mm Hg and a pronounced base deficit of more than 20 mmol/L, that continue into the early reperfusion phase (71). An experimental study in swine demonstrated that after 3 min of untreated VF arrest and 8 min of CPR, the intramyocardial pH decreased to <6.5 and the intramyocardial PCO_2_ rose above 350 mm Hg (72). As for all endogenous cases of severe acidosis, resolution and thus survival ultimately depends on reversal of the cause. In the context of CPA, resolution of acidosis requires reinstitution of adequate tissue blood flow through ROSC. However, severe acidosis alone may lead to changes in cellular metabolism that hinders reinstitution of critical myocardial function, although the relationship between pH and cellular dysfunction varies with animal species, temperature and severity and type of acidosis (73). Experimental studies have shown that low tissue pH can have profound effects on cellular function, such as reduced myocardial contractility and loss of myocardial beta-adrenoreceptor responsiveness (74–76). Based on the assumption that the extreme cellular acidosis occurring during CPA is harmful, intra-arrest buffer therapy has been widely studied in experimental animal and human studies.

Several experimental studies in dogs found a benefit of buffer administration during CPR. Sanders et al. (77) in a prolonged VF model of CPA using dogs (20–30 kg bodyweight) administered 25 mEq of NaBic prior to induction of VF, 20 mEq NaBic after 5 min of VF, 10 mEq every 5 min thereafter and 50 mEq after the first defibrillation attempt. No vasopressors or other drugs were administered, and defibrillation was attempted after 30 min of chest compressions. Bicarbonate administration improved both ROSC and survival to 24 h. Vukmir and colleague demonstrated that the benefit of NaBic varies with the duration of untreated CPA. In dogs experiencing untreated VF CPA for either 5 or 15 min, 1 mEq/kg of NaBic and subsequent additional boluses to maintain the base excess above -5 mmol/L were administered in addition to standard ALS. Buffer therapy markedly improved ROSC rates after prolonged CPA (15 min CPR) compared to ALS without NaBic but showed no benefit with short CPA (5 min CPR). In addition, CoPP in the NaBic group (35.6 ± 25.2 mm Hg) was found to be more than double that in the control group (15.3 ± 16.0 mm Hg) in the dogs with prolonged CPA (78). In a third study, NaBic administration (2 mEq/kg, once) at the beginning of CPR after 10 min of untreated VF improved ROSC and short term survival, reduced the number of required defibrillation attempts, and increased CoPP (23 ± 6 vs. 9 ± 2 mm Hg) compared to 0.9% saline in control animals (79). In a similar canine CPA model, NaBic (1 mEq/kg, once) or an equivalent dose of alternative buffer administered after 18 min of CPA (10 min of untreated VF and 8 min of CPR), improved ROSC and shortened the duration to successful defibrillation (80). But not all canine studies are supportive of buffer administration. Two additional experimental canine studies with overall shorter durations of untreated VF or CPR showed no benefit of intra-arrest buffer therapy (81, 82). A series of canine studies conducted by Bleske and colleagues did not find any benefit of buffer therapy but established that alkalemia can result from indiscriminate administration of NaBic. The CPA model used in these studies was less injurious than those presented above and animals suffered from only moderate levels of acidemia (83–85). An additional canine study demonstrated that a very large dose of NaBic (0.43 mEq/kg/min of arrest) will reliably lead to profound arterial alkalemia and hypercapnia, and a paradoxical decrease in CSF pH (see paragraph below) (86). In addition, two swine CPR studies with shorter durations of ischemic/anoxic insults failed to reveal any benefit of buffer administration (87, 88). Taken together, these experimental canine studies suggest that buffer therapy might be beneficial if applied after prolonged cardiac arrest, and thus in the presence of severe acidosis, and when used in conjunction with standard ALS treatment including vasopressors.

A major theoretical concern with NaBic-treatment of acidosis is that CO_2_ is generated as bicarbonate buffers protons. This increase in CO_2_ is reflected in a rise in PaCO_2_ and EtCO_2_ of 5–10 mm Hg for 1–3 min that can be observed after intra-arrest administration of NaBic (1–2 mEq/kg) (83, 87, 89). In contrast to bicarbonate, CO_2_ is capable of rapidly entering the intracellular space thereby “paradoxically” worsening cytosolic acidosis and potentially further disrupting cell function. However, this *in vitro* effect is of questionable relevance *in vivo* (90). Non-CO_2_ generating buffers, such as CarbiCarb (an equimolar solution of Na_2_CO_3_ and NaHCO_3_) or THAM (tromethamine, an organic amine buffer) were tested as alternatives to bicarbonate, but studies in dogs and swine did not show any benefit over NaBic (80, 87, 88). Moreover, NaBic was at least as effective in correcting cerebral pH in dogs after cardiac arrest as THAM and paradoxical cerebral acidosis was not observed with either of the compounds (91). In fact, animal studies suggest a neuroprotective effect of buffer therapy during cardiac arrest with improved cerebral perfusion, attenuated cerebral acidosis, and reduced cerebral glutamate and neuronal ischemic cell death (92, 93). Moreover, the non-CO_2_ generating buffer CarbiCarb did not improve myocardial tissue pH in swine during CPR when compared to NaBic (88).

In people, 2 RCTs and one systematic review that incorporates the findings of observational studies with a total of more than 18,000 patients showed no overall benefit of intra-arrest buffer therapy, with some studies reporting worse outcomes with buffer therapy (94–96). One RCT showed a significant benefit of NaBic administration (1 mEq/kg) in the subpopulation experiencing ALS of more than 15 min (95).

The last evidence evaluation by the International Liaison Committee on Resuscitation examining intra-arrest buffer therapy was conducted in 2010 and did not recommend routine administration of NaBic or any other buffer (46). However, the 2010 AHA guidelines recommend that in special circumstances such as hyperkalemia, severe preexisting acidemia or certain poisonings administration of NaBic (1 mEq/kg IV) can be considered, while care must be taken to avoid potential adverse effects such as hypernatremia or iatrogenic alkalemia (97). For dogs and cats, the 2012 RECOVER guidelines recommend that NaBic administration (1 mEq/kg IV) may be considered in addition to standard ALS in animals when CPA continues for more than 10–15 min (1).

## Author Contributions

DF and MB co-wrote and critically revised the manuscript. Both authors read and approved the final manuscript.

## Conflict of Interest

The authors declare that the research was conducted in the absence of any commercial or financial relationships that could be construed as a potential conflict of interest. The reviewer SD declared a past co-authorship with one of the authors (DF) to the handling Editor.

## Abbreviations

ADP: aortic diastolic pressure
ALS: advanced life support
BLS: basic life support
CCB: calcium channel blocker
CePP: cerebral perfusion pressure
CoPP: coronary perfusion pressure
CPA: cardiopulmonary arrest
CPR: cardiopulmonary resuscitation
CVP: central venous pressure
ETCO2: end tidal CO2
HES: hydroxyethyl starch
HHS: hypertonic saline/hydroxyethyl starch combination
HS: hypertonic saline 7.2%
IHCA: in-hospital cardiac arrest
ICP: intracranial pressure
LAST: local anesthetic systemic toxicity
LVEDP: left ventricular end-diastolic pressure
LVEDV: left ventricular end-diastolic volume
MAP: mean arterial pressure
NaBic: sodium bicarbonate
OHCA: out-of-hospital cardiac arrest
RECOVER: Reassessment Campaign on Veterinary Resuscitation
RCT: randomized controlled trial
ROSC: return of spontaneous circulation
SD: standard deviation
VF: ventricular fibrillation.
